# A Mobile Health Team Challenge to Promote Stepping and Stair Climbing Activities: Exploratory Feasibility Study

**DOI:** 10.2196/12665

**Published:** 2020-02-04

**Authors:** Seaw Jia Liew, Alex Wilhelm Gorny, Chuen Seng Tan, Falk Müller-Riemenschneider

**Affiliations:** 1 Saw Swee Hock School of Public Health National University of Singapore National University Health System Singapore Singapore; 2 Yong Loo Lin School of Medicine National University of Singapore National University Health System Singapore Singapore; 3 Institute of Social Medicine, Epidemiology and Health Economics, Charité - Universitätsmedizin Berlin Germany

**Keywords:** behavior, health, physical activity, wearables

## Abstract

**Background:**

Mobile health (mHealth) approaches are growing in popularity as a means of addressing low levels of physical activity (PA).

**Objective:**

This study aimed to determine the validity of wearables in measuring step count and floor count per day and assess the feasibility and effects of a 6-week team challenge intervention delivered through smartphone apps.

**Methods:**

Staff and students from a public university were recruited between 2015 and 2016. In phase 1, everyone wore a Fitbit tracker (Charge or Charge HR) and an ActiGraph for 7 days to compare daily step count estimated by the two devices under free-living conditions. They were also asked to climb 4 bouts of floors in an indoor stairwell to measure floor count which was compared against direct observation. In phase 2, participants were allocated to either a control or intervention group and received a Fitbit tracker synced to the Fitbit app. Furthermore, the intervention group participants were randomized to 4 teams and competed in 6 weekly (Monday to Friday) real-time challenges. A valid day was defined as having 1500 steps or more per day. The outcomes were as follows: (1) adherence to wearing the Fitbit (ie, number of days in which all participants in each group were classified as valid users aggregated across the entire study period), (2) mean proportion of valid participants over the study period, and (3) the effects of the intervention on step count and floor count determined using multiple linear regression models and generalized estimating equations (GEEs) for longitudinal data analysis.

**Results:**

In phase 1, 32 of 40 eligible participants provided valid step count data, whereas all 40 participants provided valid floor count data. The Fitbit trackers demonstrated high correlations (step count: Spearman ρ=0.89; *P*<.001; floor count: Spearman ρ=0.98; *P*<.001). The trackers overestimated step count (median absolute error: 17%) but accurately estimated floor count. In phase 2, 20 participants each were allocated to an intervention or control group. Overall, 24 participants provided complete covariates and valid PA data for analyses. Multiple linear regressions revealed that the average daily steps was 15.9% higher for the intervention group (95% CI −8.9 to 47.6; *P*=.21) during the final two intervention weeks; the average daily floors climbed was 39.4% higher (95% CI 2.4 to 89.7; *P*=.04). GEE results indicated no significant interaction effects between groups and the intervention week for weekly step count, whereas a significant effect (*P*<.001) was observed for weekly floor count.

**Conclusions:**

The consumer wearables used in this study provided acceptable validity in estimating stepping and stair climbing activities, and the mHealth-based team challenge interventions were feasible. Compared with the control group, the participants in the intervention group climbed more stairs, so this can be introduced as an additional PA promotion target in the context of mHealth strategies. Methodologically rigorous studies are warranted to further strengthen this study’s findings.

## Introduction

### Background

Low levels of physical activity (PA) can be attributed to societal development and modern workplaces, schools, and homes [1,2]. Physical inactivity has been identified as one of the leading risk factors for noncommunicable diseases, including obesity, diabetes, and heart diseases, and as a cause of preventable death [3,4]. Studies have demonstrated that even light PA can confer relevant health benefits among inactive individuals [5-11]. Simple measures that increase activities of daily living (eg, steps walked and stairs climbed) can be incorporated into daily life in public and work settings to provide health benefits [12-14]. Although stepping activities are a widely used target for PA promotion strategies [15,16], stair climbing has been identified as an actionable and time-saving approach; it is usually of greater intensity (ie, moderate-to-vigorous PA, MVPA) [17] and has been found to confer health benefits, such as improvements in cardiorespiratory fitness [18,19] and blood pressure [13]. Stair climbing and strategies to promote stair climbing may also be particularly appropriate among adults in Asia and metropolitan areas, which are densely populated with many high-rise buildings. Point-of-decision prompts have been widely used to encourage stair climbing at the population level [20]. However, in the long term, these strategies have not proven effective because people may have become habituated to these cues over time [21].

Today, electronic health and mobile health (mHealth) technologies (eg, wireless communication technologies, database server, sensors embedded in smartphone and wearable devices, and smartphone apps) enable us to objectively collect continuous PA and other contextual information under free-living conditions (ie, usual daily activities carried out in the community or at home). Moreover, improved design and enhanced digital connectivity have allowed more interactive and entertaining strategies for behavioral change to be developed [16,22-26]. For example, modern PA wearables and smartphone apps provide users with quantitative visual feedback on their PA level and empower them to continuously monitor and improve their behavior. Behavior change techniques (BCTs), such as goal setting, self-monitoring, and feedback, have been found effective in health promotion [27-29]. Moreover, gamified interventions have been shown to further improve user engagement and thereby potentially increase intervention effectiveness [30,31]. Examples include leaderboards, team-based performance feedback, or social and financial incentives, which have been shown to translate to better PA outcomes [32-34]. Feedback is a common BCT that can improve goal attainment through action planning [35] by giving explicit suggestions on when, where, and how to perform the action [36]. In addition to the continuous objective monitoring of diverse sensor-based information, mHealth technologies also enable the provision of continuous feedback in real time, which can be effectively integrated into daily routines and thus potentially increase the effectiveness of PA interventions in real-life settings.

Systematic reviews suggest that using pedometers paired with goal setting increased daily step count and resulted in decreases in body mass index and systolic blood pressure [15]. In the advent of consumer PA wearable devices beyond pedometers, there has been an increasing number of studies that investigated the effects of these wearables on promoting PA levels, in part with improvements in step count [37]. The potential of mHealth interventions on promoting stair climbing remains unstudied, despite the potentially greater health impact of this more intensive form of PA. With regard to the accuracy of consumer wearables to measure PA-related outcomes, previous validation studies have suggested that step count information from such devices may correlate well with established measures, but they may also under- or overestimate activity levels in absolute terms, which is an important consideration in setting appropriate goals or incentives [38,39]. The literature also suggests that the accuracy of different types of wearables differs considerably [38,40] and that fewer studies were conducted in free-living settings [38,41,42]. To the best of our knowledge, no published study has assessed consumer devices with regard to their accuracy of measuring stair climbing activities. This warrants further investigation before stair climbing activities can be incorporated as a target for mHealth interventions, aiming to promote PA.

### Objectives

This feasibility study aimed to address some of these evidence gaps to guide the development of large-scale studies and health promotion initiatives in the future. The study included an assessment of the validity and feasibility of using an mHealth technology suite for the implementation of a real-time PA intervention to increase stepping and stair climbing activities among adults in the general population. The intervention to be tested comprised a team challenge, using a random team-forming approach to overcome two key limitations of team-based interventions: (1) scalability because of the need to identify and register a team, and (2) biases in findings when participants self-select team members [43]. The specific aims of this study were (1) to develop a multicomponent mHealth technology suite, which comprised PA wearables (ie, Fitbit Charge and Charge HR) and an interactive smartphone app supported by a Web-based data management system, (2) to determine the validity of these integrated wearables in measuring number of steps taken per day and number of floors climbed per day, and (3) to assess the feasibility and explore the effects of the team challenge intervention (ie, a pilot controlled trial) targeting adults at work or in a tertiary education setting from Monday to Friday. Leveraging several key BCTs [44], the team challenge adopted gamification features, such as feedback, social support, social incentive or reward, and self-monitoring, to facilitate PA behaviors.

## Methods

### Study Participants

Recruitment of participants was conducted through posters, dissemination of e-flyers, and emails detailing the study objectives and inclusion and exclusion criteria through 4 faculties at the National University of Singapore from 2015 to 2016. Staff or students were deemed eligible if they (1) were aged 21 to 65 years, (2) free from physical disabilities or illnesses that would restrict normal activities, (3) were English literate, and (4) owned a smartphone that could support the use of a Fitbit tracker (Fitbit Charge or Charge HR). Potential participants were excluded if they intended to travel abroad for the duration of the study. Participants provided informed consent to participate in the study. The National University of Singapore Institutional Review Board approved the study protocol.

### Study Design

**Figure 1:**
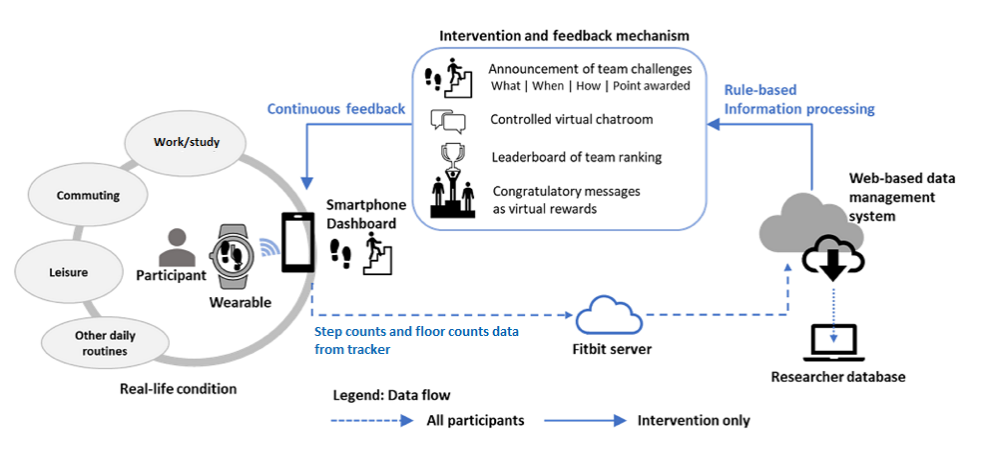
Internet and mobile health technologies to deliver team-based physical activity intervention and enhance user experience.

**Figure 2:**
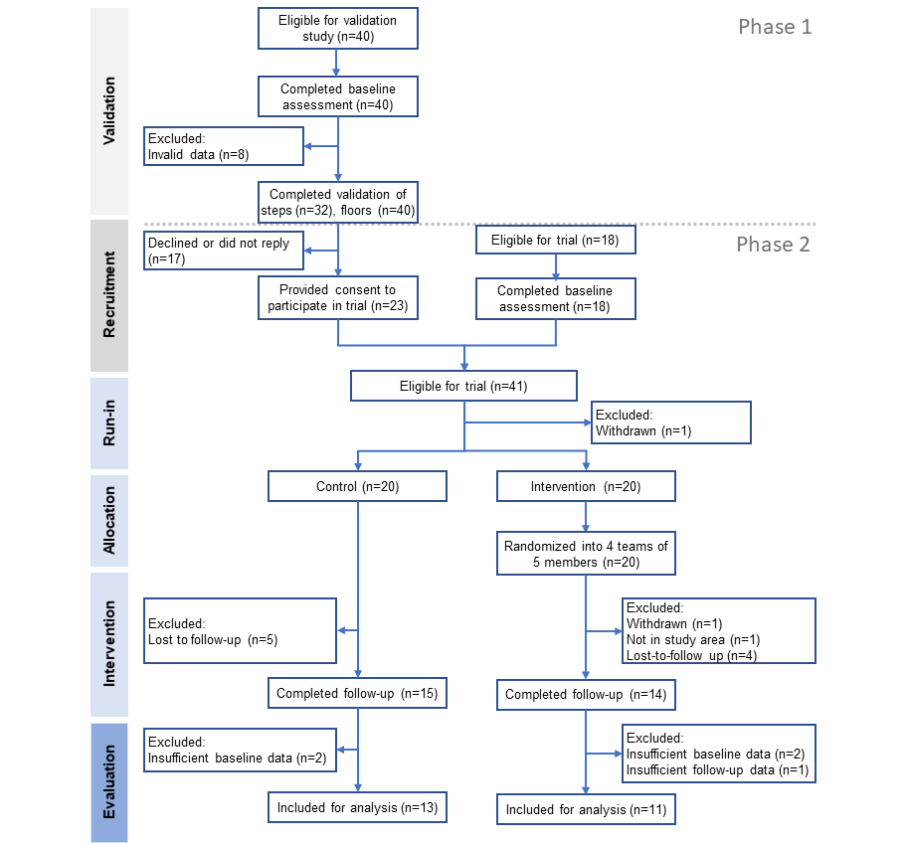
Participant flow diagram.

This pilot study involved 3 sequential processes: (1) conceptualization of team challenge intervention delivered through a purposively developed multicomponent mHealth technology suite, which comprises consumer PA wearables, researcher-designed smartphone apps integrated with interactive gamification features, and a Web-based data management system as shown in Figure 1; (2) a validation study to determine the validity of the Fitbit wearables in measuring daily steps (ie, a step refers to lifting one’s foot and putting it down in a different place) and floor count (ie, 1 floor count is equivalent to a 10 feet upward vertical displacement); and (3) a feasibility study (ie, a controlled pilot trial) that aimed to evaluate the real-time team challenge intervention integrated in the purposively developed mHealth system.

### Mobile Health Technology Suite

Every participant recruited for the feasibility study was provided with a wearable device (either Fitbit Charge or Charge HR tracker at random) and the Fitbit proprietary app. However, only intervention group participants were registered for the additional use of the mHealth technology suite (Figure 1). The mHealth suite was realized through an integration of 3 major components: (1) Fitbit tracker and its proprietary app, (2) a purposively developed smartphone app with interactive functionalities, and (3) a Web-based data management unit. Fitbit was chosen for this study given the relative popularity the brand enjoyed at the time this pilot study was conducted. To complement the Fitbit tracker and the proprietary Fitbit app, a separate smartphone app and a Web portal compatible with the iOS and Android mobile systems were developed and linked with the Fitbit server through the application programming interface (API) provided by Fitbit. Furthermore, the apps sent push notifications and delivered in-app team challenges that aimed to engage intervention group participants in taking more steps and stairs every day. All smartphone apps and Web portal were password protected, and participants were asked not to input any personal information for the duration of the study. The data were downloaded from the Web portal by the research team at the end of the study.

### Intervention

#### Team Formation

The intervention group participants were randomly assigned to smaller teams to avoid undue advantages of one team over the other. This study tested a team-based approach, which could potentially be replicated in large-scale settings (1) to promote team-based PA yet prevent high disparities in PA levels among teams; (2) to encourage PA and facilitate team forming in a real-world situation, especially in workplace or school settings when a person’s PA buddies may not always be around in the same geographical locations; and (3) to create an avenue for individuals with a restricted social network to attempt team-based activities without the hassle of forming a physical team. The concept of anonymous sign up (nonregistering) of teams was implemented and tested in this pilot study because it helps to overcome the following two key limitations of commonly used team-based challenges: (1) scalability because of the need to identify and register a team, and (2) biases related to self-selection of teams and team members [43].

#### Team-Based Challenges

The intervention comprised team-based challenges with multiple components (Textbox 1). Daily stepping and stairs climbed were quantified as the number of steps taken per day and the number of floors climbed per day, respectively. The challenges were designed to run on weekdays (from Monday to Friday) exclusively during the intervention period. All intervention group participants were randomly allocated into teams of equal size and prompted via smartphone push notifications to participate in the challenges. The intervention lasted 6 weeks, comprising repeated team-based challenges (weeks 1 and 4: steps challenge, weeks 2 and 5: stair climbing challenge, and weeks 3 and 6: steps challenge + lunchtime dash) that were prescheduled on a weekly basis. The lunchtime dash was a variation in which additional points were given for increase in step count and floor count during lunch hour.

**Features of the team challenge and purposively developed smartphone app.**

Time of challenge: weekdays (Monday to Friday) 12 AM to 11:59 PM

Duration of challenge: 6 weeks

Types of challenges

• Weeks 1 and 4: steps challenge

• Weeks 2 and 5: stair climbing challenge

• Weeks 3 and 6: steps challenge + lunchtime dash (12 PM to 3 PM; additional points were awarded for steps taken and floors climbed during lunchtime)

Delivery of intervention: purposively developed smartphone app with interactive in-app features

• Prescheduled announcement of challenge: type, time, duration, and points awarded

• Virtual live chat: controlled message exchange among team members

• Leaderboard: visual feedback of weekly team ranking

• Virtual rewards: congratulatory message to the winning team

#### Gamification

Gamification (Multimedia Appendix 1) was adopted to motivate teams to compete to achieve goals and create a platform for team members to review their PA performance, communicate, and establish peer support to earn rewards for the team. In this study, in addition to delivering challenge-related notifications, the study app was developed to provide gamified features available only to the intervention group. To experience these gamification features, notifications were sent during the first 2 weeks of the intervention to remind the intervention group participants to synchronize their trackers with the study app. An individual’s PA measures were converted to the team’s performance scores.

#### Prescheduled Challenge Announcements

Actionable feedback was realized by providing explicit information about the weekly challenges: what (type of challenges; ie, stepping, stair climbing, or lunchtime dash challenge), when (dates and time of weekly challenge), how (type of PA and measures, such as step count or floor count), and reward (the associated scoring scheme and awarded points for every increase in the number of steps taken or floors climbed).

#### Real-Time Feedback

Team-level feedback on PA performance (daily step count or floor count) were delivered through weekly push notifications and also reflected on the leaderboard. An individual’s PA statistics in terms of steps taken and stairs climbed were delivered in real time through the study app.

#### Virtual Chatroom

A virtual chatroom with an instant messaging feature was made available to all competing teams, but the exchange of short messages was restricted to only team members. Teammates remained anonymous throughout the study, and the exchange of messages was moderated by the research team.

#### Leaderboard

The leaderboard, a visual feedback of weekly team rankings, was made available to all intervention group participants.

#### Virtual Rewards

The incentives administered through our system were nonmaterial in nature. At the end of each challenge week, congratulatory messages were delivered to the winning team, and the final team ranking for the week was updated on the leaderboard.

### Study Procedure

The study was organized into the following two main phases: (1) a validation study and (2) a feasibility study, that is, the evaluation of the controlled pilot trial, which consisted of the run-in (baseline), intervention, ending, and postintervention phases, as defined in Table 1.

**Table 1:** Definition of study phases in temporal order.

Study phase		Definition
**Phase 1: validation study**		
	Validation of daily steps	7 days of wearing physical activity wearables in free-living conditions Fitbit-measured step count data were compared with those measured using ActiGraph
	Validation of floor count	4 bouts of upward stair climbing: 10 ft, 20 ft, 10 ft, and 20 ft in indoor stairwell (10 ft upward vertical displacement is equivalent to 1 floor count) Fitbit-measured floor count data were compared with visual observation
**Phase 2: feasibility study**		
	Run-in (baseline)	10 days (including weekdays and weekends) before group allocation when baseline step count and floor count measurements were established
	Intervention	6 weeks of intervention (comprising 6 weekly challenges running from Monday to Friday) or 6 weeks of free living for the control group
	Ending	Last 10 weekdays (Monday to Friday of week 5 and week 6) of the intervention phase
	Postintervention	5 weekdays after the intervention phase

#### Phase 1: Validation Study

Daily steps validation was conducted in free-living conditions for 7 consecutive days, whereas floor count validation involved 4 bouts of upward stair climbing in an indoor stairwell. Details on recruitment have been published in a previous paper [38]. Briefly, members of the university’s staff and students were recruited through department-approved internal emails. All participants self-reported sociodemographic information through a Web-based questionnaire and completed measurements of height and weight necessary to initialize the devices used in the study. Each participant received a password-protected Fitbit mobile app, a Fitbit account that was identified using a unique subject identification number, and a wrist-worn Fitbit Charge or Charge HR that was linked to the Fitbit account. Participants were allowed to view their PA data from the Fitbit official dashboard, and they were advised to charge the wearable regularly. Subsequently, participants were brought to an indoor stairwell to validate the floor count function of the device. Each participant embarked on 4 separate bouts of climbing (upward): 10 ft, 20 ft, 10 ft, and, finally, 20 ft of vertical height, giving rise to a cumulative height climbed of 60 ft (ie, 10 ft is equivalent to 1 floor count) per person. Total floor count after each bout of climbing was read off the Fitbit display. Following stair climbing, participants were asked to wear an ActiGraph GT3X+ accelerometer on the right hip, concurrently with the wrist-worn Fitbit tracker, to provide 7 days’ worth of steps data during waking hours. All participants received prompts to record their time of accelerometer use through a smartphone-based activity diary.

The ActiGraph-measured step count data in 60-second epochs were downloaded using ActiLife 6 software (ActiGraph, LLC), whereas the Fitbit-measured step count data were extracted from the Fitbit Web server using a developer’s API issued by Fitbit. A valid day was defined as having accumulated 1500 steps or more taken per day [45] with 10 hours or more per day, restricted only to common wear time based on both ActiGraph and Fitbit tracker. Participants who provided 4 or more valid days [46] of ActiGraph and Fitbit data were included for the validation analysis.

### Phase 2: Feasibility Study

The feasibility study was a controlled pilot trial with the following objectives: (1) to determine adherence to wearing the Fitbit (ie, number of days in which all participants in each group were classified as valid users aggregated across entire study period), (2) to determine the mean proportion of valid participants over the study period, and (3) to explore the effects of the team challenge intervention in promoting stepping and stair climbing among the completers (ie, participants who provided complete baseline information and valid Fitbit data at both baseline and ending phases). Recruitment was open to the existing participants who had completed the validation study as well as new participants. Newly recruited participants were also asked to complete the baseline questionnaire, and their height and weight were measured. All eligible participants entered the run-in (baseline) phase, which allowed new participants to familiarize themselves with using the Fitbit trackers and Fitbit app. All participants were assisted during the installation of the purposively developed study app at baseline. Despite this assistance, it was not possible to successfully install and enable the latest version of the study app on iOS devices and certain Android phones because of unresolvable incompatibility issues. As such, participants who successfully used the purposively developed study app were allocated to the intervention group. Participants who could not use the study app because of incompatibility were assigned to the control group. During the run-in period, app features (ie, team challenge, leaderboard, virtual chatroom, and reward) were not activated. After group allocation was determined, the intervention started for all intervention group participants at the same time. Both groups were then followed up for 6 weeks with an additional 1 week of free living (postintervention).

PA was measured using the Fitbit devices as the number of steps taken per day or the number of floors (10 ft) climbed per day. Baseline PA was quantified as the average number of steps taken per day or number of floors (10 ft) climbed per day during the run-in period. Ending PA was quantified as the average number of steps taken per day or number of floors (10 ft) climbed per day during the last 10 weekdays (ie, Monday to Friday on week 5 and week 6) of the 6-week intervention period. The main outcome was the difference between the intervention and control groups in terms of PA data from baseline to the ending phase. Additional outcomes were as follows: (1) adherence to Fitbit use (ie, number of days in which all participants in each group were classified as valid users aggregated across entire study period), and (2) the mean proportion of valid participants over the study period.

A valid day was defined as having accumulated 1500 steps or more per day [45]. Step count and floor count data from invalid days were not considered. Only participants who achieved 4 or more valid days of data [46] at both run-in (baseline) and ending phases were considered valid users and included in the analyses. On the basis that the team challenges were conducted on weekdays (ie, from Monday to Friday), the estimation of potential intervention effects used only PA data from weekdays.

### Statistical Analysis

#### Phase 1: Analyses of Data From Validation Study

For the validation of daily steps taken per day, the assessment of the convergent validity of Fitbit compared with the ActiGraph was done on a day-to-day basis by aggregating the number of steps taken within each day. Spearman correlation coefficient (ρ) and intraclass correlation coefficient (ICC) were used to assess correlation and agreement, respectively, in step count data between ActiGraph and Fitbit. The Spearman ρ ranges from −1 (perfect negative correlation) to +1 (perfect positive correlation). An absolute Spearman correlation magnitude of 0.90 to 1.00 implied very high, 0.70 to 0.89 implied high, 0.50 to 0.69 implied moderate, 0.30 to 0.49 implied low, and 0.30 or less implied negligible correlation [39]. The agreement between measurements was interpreted based on the ICC estimate: greater than 0.90 implied excellent, 0.75 to 0.90 implied good, 0.50 to 0.75 implied moderate, and less than 0.50 implied poor agreement [40]. The median of absolute percentage error (MdAPE) between step count measured by Fitbit and ActiGraph was defined as the median of:

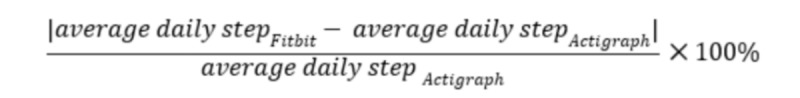



For the validation of floor count, the Spearman ρ and ICC estimate were used to assess correlation and agreement, respectively, in floor count data between visual observation and Fitbit. The MdAPE between floor count data obtained by Fitbit and visual observation was defined as the median of:

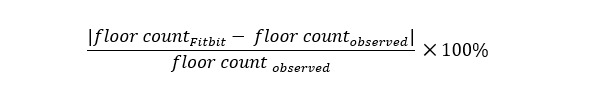



#### Phase 2: Analyses of Data From Feasibility Study

Binary baseline characteristics of completers were compared with noncompleters using Fisher exact test with a 2-sided *P* value and the prevalence ratio. Among the completers, comparisons between intervention and control groups were performed using Fisher exact test for categorical variables and Wilcoxon rank-sum test for continuous variables, where a 2-sided *P* value was reported for both tests.

#### Adherence

The proportion of valid participants on each observation day was defined by the number of participants who contributed valid step data on the day divided by the total number of participants (according to group). The adherence of each group was determined by calculating the rate ratio (RR) of the mean proportion of each group across the 40-day observation period. The difference in the mean proportion of valid users between the intervention and control groups was assessed using 2 independent sample *t* test.

Stepping and stair climbing activities were described as the average number of steps taken per day and average number of floors (10 ft) climbed per day, respectively. PA-related variables (ie, steps and floors) were log transformed to reduce their skewness and approximate normal distributions.

#### Intervention Effect at Ending Phase

The intervention effects between the intervention and control groups at the ending phase were estimated from multiple linear regression analyses on log-transformed average number of steps taken per day and log-trasnformed average number of floors (10 ft) climbed per day, respectively, with adjustment for baseline covariates (ie, gender, age, and respective baseline PA variables). After fitting the multiple linear regression models, the model assumptions were checked with residual diagnostics. The intervention effects were reported in terms of percent change in the average number of steps taken per day or average number of floors (10 ft) climbed per day between the 2 groups with the control group as the reference.

#### Intervention Effect at Each Challenge Week

Generalized estimating equations (GEEs) analyses were performed on the log-transformed average number of steps taken per day and log-transformed average number of floors (10 ft) climbed per day for each week during the intervention period to detect potentially different weekly intervention effects by taking into account the within-subject correlation of repeated weekly outcomes from the same individual. The multiple linear regression model with GEE was performed where an autoregressive working correlation matrix was specified, and the Huber-White estimator of variance was used to obtain a robust estimate for the standard error. We assessed the interaction between group status (ie, intervention group vs control group) and intervention week (ie, week 1 to week 6) with adjustment for baseline covariates (ie, gender, age, and respective baseline PA variables). When the interaction term was significant, a week-specific intervention effect was reported. On the other hand, if the interaction term was not significant, we reported the model that did not include the interaction term, and a common intervention effect across 6 weeks was reported instead. The model assumptions were checked with residual diagnostics. The intervention effects were reported in terms of percent change in the average number of steps taken per day or number of floors (10 ft) climbed per day for each week compared with the control group.

This study was a feasibility study. Thus, sample size calculation was not performed. All statistical analyses were conducted using STATA 14.0 for Windows (Stata Corporation).

## Results

### Participants’ Characteristics

Participants were staff and students from the National University of Singapore, recruited between 2015 and 2016 (Figure 2). A total of 40 eligible participants provided informed consent to participate in the validation study and completed the baseline assessment. In the phase 1 validation study, 80% (32/40 eligible participants) completed daily steps validation, whereas 100% (all 40 eligible participants) completed floor count validation. Of 40 participants, 23 (58%) further consented to participate in the subsequent feasibility study (phase 2). In addition, 18 participants were recruited, resulting in 41 eligible individuals for phase 2. Of the eligible participants, 1 participant subsequently declined participation, and 40 participants who completed the run-in period were allocated to the intervention (n=20) and control (n=20) groups. Of 40 participants, 29 (73%) completed the 6-week follow-up but 24 (60%; intervention group=11 and control group=13) participants were classified as completers if they provided complete exposure data and valid PA data at both baseline and ending phases. These 24 completers were included in the analyses of intervention effectiveness. Baseline characteristics of participants involved in the validation study and feasibility study are summarized in Table 2. No significant differences in the baseline characteristics were observed between the completers and the noncompleters in this study population (Multimedia Appendix 2).

**Table 2:** Baseline characteristics of participants in validation (n=40) and pilot trial (n=24) included in analyses.

Baseline characteristics		Phase 1: validation study (N=40)	Phase 2: feasibility study (N=24)		
			Intervention group (n=11)	Control group (n=13)	*P* value^a^
Age (years), median (25th to 75th percentile)		24 (23-30)	28 (23-35)	28 (23-31)	.79
**Gender, n (%)**					**.03**
	Female	20 (50)	4 (36)	11 (85)	
	Male	20 (50)	7 (64)	2 (15)	
**Ethnicity, n (%)**					**.30**
	Chinese	33 (82)	8 (73)	12 (92)	
	Non-Chinese	7 (17)	3 (27)	1 (8)	
**Education, n (%)**					**.66**
	Secondary^b^	15 (36)	4 (36)	3 (23)	
	Tertiary or above	25 (62)	7 (64)	10 (77)	
**Work status, n (%)**					**>.99**
	Studying	27 (67)	6 (54)	7 (54)	
	Working	13 (32)	5 (45)	6 (46)	
**Marital status, n (%)**					**.63**
	Not married	37 (92)	8 (73)	11 (85)	
	Married	3 (7)	3 (27)	2 (15)	
**Body mass index (kg/m^2^), n (%)**					**.14**
	<18.5	1 (2)	0 (0)	2 (15)	
	18.5-22.9	26 (65)	8 (73)	7 (54)	
	23.0-24.9	7 (17)	1 (9)	4 (31)	
	≥25.0	6 (15)	2 (18)	0 (0)	

^a^In Phase 2, feasibility study: for continuous variables, comparisons were performed by Wilcoxon rank-sum tests (medians); for categorical variables, Fisher exact tests were used.

^b^Secondary educational level included participants who completed A level or attended polytechnic school.

### Results of Validation Study

A total of 32 participants provided 215 valid step count data pairs (measured by both ActiGraph and Fitbit) with an average of 6.7 (SD 0.6) valid data pairs. In the floor count validation study, each of the 40 participants provided 1 valid floor count data pair (direct observation vs measured by Fitbit; Table 3). A high and very high positive correlations between measurements in step and floor counts were observed, respectively. The level of agreement was good for number of steps taken per day and excellent for floor count. The MdAPE was less than 20% for step count and zero for floor count.

**Table 3:** Correlation, agreement, and error in measurements of steps (ActiGraph vs Fitbit) and floors (observation vs Fitbit).

Measurement characteristics		Daily steps^a^ (n=32)	Floor count^b^ (n=40)
Setting		7 days, free-living conditions	6 floors, research setting
Number of valid data pairs^c^		215	40
Number of valid data pairs provided by each participant, mean (SD)		6.7 (0.58)	4 (0)
**Measurements output, median (25th-75th percentile)**			
	ActiGraph or observation	9503 (6392-12,479)^d^	6 (6-6)
	Fitbit-measured	11,148 (8186-14,493)	6 (6-6)
**Difference in measurements, median (25th-75th percentile)**			
	Fitbit vs ActiGraph or observation	1398 (643-2720)	0 (0-0)
Spearman correlation (ρ)		0.89^e^	0.98^e^
Intraclass correlation^f^ (95% CI)		0.81 (0.45 to 0.91)^e^	0.96 (0.95 to 0.97)^e^
Median absolute percent error, median (25th-75th percentile)		17.13 (7.80-30.29)	0 (0-0)

^a^Daily steps were presented as the number of steps taken per day.

^b^One floor count is equivalent to 10 ft upward vertical displacement (presented as the number of floor).

^c^Valid data pairs referred to valid data points provided by both ActiGraph and Fitbit.

^d^ActiGraph-measured steps (presented as the number of steps taken per day).

^e^*P*<.001.

^f^Intraclass correlation derived using 2-way mixed effects model for absolute agreement.

#### Wearable Use During the Study Period

**Figure 3:**
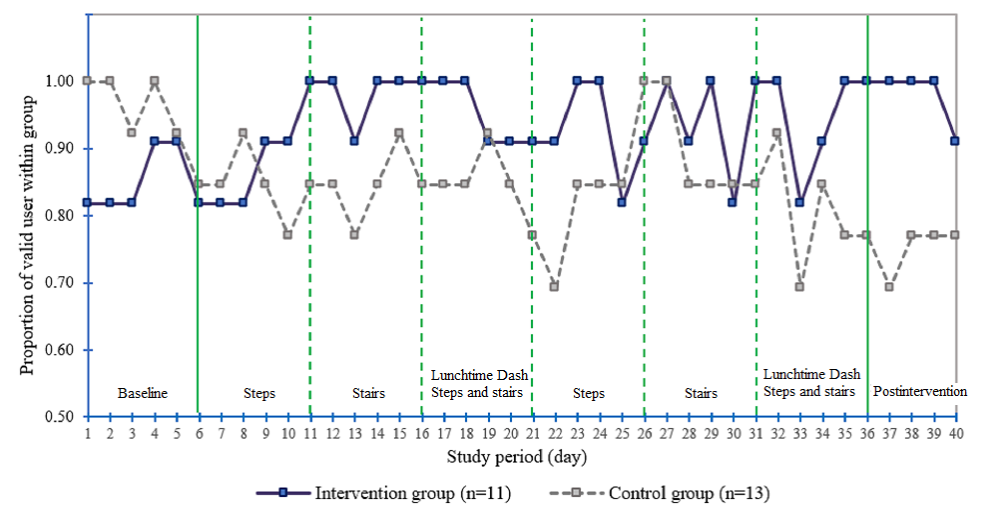
Proportion of valid users (intervention, n=11; and control, n=13) over study phases.

The proportion of valid users over the 40-day study period within each group is described in Figure 3. At baseline, the proportion of valid users was somewhat higher among the controls; however, it decreased over time. On the contrary, the proportion of valid users in the intervention group was lower at baseline, but it increased after the introduction of the first team challenge and remained largely consistent throughout the study period. Over the 40 days of observation, the intervention group participants demonstrated a higher level of Fitbit use adherence. All intervention participants (11/11) were classified as valid in 18 of 40 days, whereas all control group participants (13/13) were valid participants in only 5 of 40 days (RR 3.6, 95% CI 1.5 to 9.1; *P*=.003). There was also a significant difference in the mean proportion of valid users between intervention and control groups (0.92 vs 0.83; *P*<.001).

#### Effects of the intervention

Table 4 presents the effects of the intervention on the average number of steps taken per day or number of floors (10 ft) climbed per day at the ending phase after adjusting for baseline PA variables, age, and gender. On average, the intervention group achieved 15.9% higher average number of steps taken per day (95% CI −8.9 to 47.6; *P*=.21) and 39.4% higher average number of floors (10 ft) climbed per day (95% CI 2.4 to 89.7; *P*=.04) than the control group at the ending phase.

**Figure 4:**
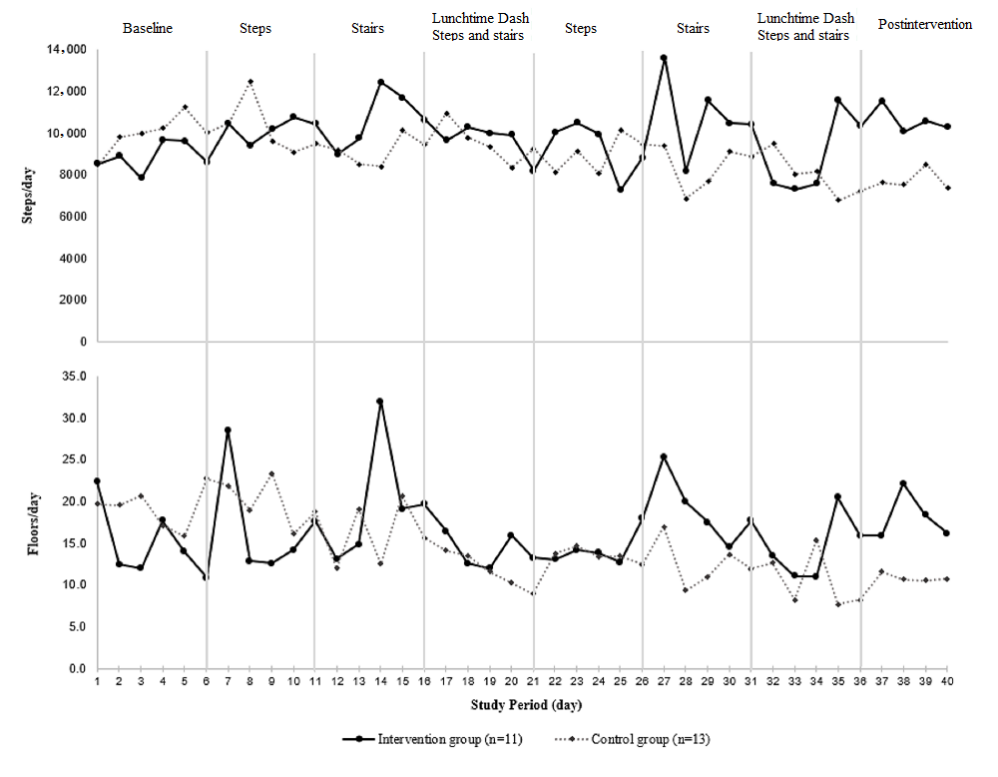
Average number of steps taken per day or number of floors (10 ft) climbed per day at baseline and across the 6-week intervention period.

Figure 4 depicts the average number of steps taken per day (upper panel) and number of floors (10 ft) climbed per day (lower panel) of the intervention period according to allocated group. The GEE results (Table 5) indicated that, on the one hand, there was no significant interaction effect between group status and intervention week with regard to average number of steps taken per day. Hence, the weekly intervention effect was similar across all 6 weeks (percentage change 9%; 95% CI −7.3 to 28.1; *P*=.30). On the other hand, a significant interaction between group status and intervention week (*P*<.001) was observed with regard to the average number of floors (10 ft) climbed per day. The percent change in the intervention group was significantly higher (percentage change 78.5%; 95% CI 20.8 to 163.8; *P*=.004) at week 5 (ie, the second stair climbing challenge) when compared with the control group.

**Table 4:** Average stepping and stair climbing activity of completers (n=24) as well as their intervention effect (percentage change).

Type of physical activity		Physical activity level by group, median (25th-75th percentile)		Intervention effect, (with respect to the control group)	
		Intervention group (n=11)	Control group (n=13)	Percentage change^a^, (95% CI)	*P* value
**Stepping activity (average number of steps taken per day)**				**15.9 (−8.9 to 47.6)**	**.21**
	Baseline	10,579 (8375-11,314)	10,207 (7745-12,345)		
	Ending phase	8797 (8124-13,181)	8881 (7828-10,375)		
**Stair climbing activity (average number of floors [10 ft] climbed per day)**				**39.4 (2.4 to 89.7)**	**.04**
	Baseline	11.8 (8.5-21.5)	14.8 (10.2-29.2)		
	Ending phase	13.6 (9.9-23.3)	13.4 (10.3-14.4)		

^a^Multiple linear regressions adjusted for its baseline physical activity, age, and gender.

**Table 5:** Generalized estimating equation results for the average number of steps taken per day or number of floors (10 ft) climbed per day across each week during the 6-week intervention.

Physical activity			Intervention effect (with respect to the control group)					
			Percentage change^a^		95% CI		*P* value^b^	
**Stepping activity (weekly average number of steps taken per day)**								
	**Weekly challenge**							
		Common effect across all weeks		9.0		−7.3 to 28.1		.30
**Stair climbing activity (weekly average number of floors climbed per day)**								
	**Weekly challenge**							
		Week 1 (steps)		−6.3		−35.5 to 36.2		.73
		Week 2 (stairs)		18.2		−15.1 to 64.5		.32
		Week 3 (steps+stairs)		24.9		−20.4 to 95.8		.33
		Week 4 (step)		15.1		−23.5 to 73.2		.50
		Week 5 (stairs)		78.5		20.8 to 163.8		.004
		Week 6 (steps+stairs)		25.3		−8.3 to 71.1		.16

^a^Generalized estimating equation: (family: Gaussian; link: identity) analyses adjusted for its baseline physical activity, age, and gender.

^b^The interaction term (Group x week) has a *P* value of 0.12 and <.001 for stepping and stair climbing activities, respectively

## Discussion

### Principal Findings

This study describes the development of an mHealth-based team challenge intervention, including the validation of the integrated wearables with regard to the 2 main PA outcome measures: stepping and stair climbing activities. It also provides initial results from a controlled pilot trial on the feasibility and effectiveness of this real-time team challenge intervention when implemented among students and staff of a public university in Singapore. Findings from this study could inform large-scale mHealth strategies, such as the Singapore National Steps Challenge [47], on the potential integration of the team-based model and the focus on stair climbing in addition to stepping activities.

In summary, the validation study demonstrated that the Fitbit trackers provided acceptable validity in estimating daily step count and floor count, although the trackers overestimated step count in absolute terms. The controlled pilot trial demonstrated that the multicomponent mHealth technology suite designed to deliver real-time interventions was feasible. About 60% of the participants continued using the system and provided valid data for evaluation. Higher adherence was observed in the intervention group, and the team challenges resulted in more PA, especially stair climbing, when compared with the control group.

Considering the rapid innovations and adoption of consumer wearables and smartphone apps in monitoring PA behaviors, these mobile technologies require formal assessments to strengthen scientific basis and justify their application in measuring PA outcomes, such as the number of steps and flights of stairs. In terms of stepping activity under free-living conditions, we observed a high positive correlation and good agreement between the consumer wearables and a validated research grade accelerometer [48]. Consistent with a study that had systematically reviewed the accuracy of various Fitbit models, the Fitbit trackers used in this study demonstrated a similar tendency of overestimating daily steps and generated a measurement error of more than 10% [38]. In contrast, the review did not identify any study that has investigated the validity of Fitbit trackers on measuring stair climbing. Our study found a very high, positive correlation and excellent agreement between Fitbit-measured floor count and direct observation. This finding adds to the current literature that these consumer wearables accurately estimate stair climbing activities, offering additional value in PA continuous monitoring beyond stepping.

Stepping, a form of PA that often occurs at a lower intensity, has been a common target of mHealth interventions. However, fewer mHealth interventions using continuous monitoring via wearables or smartphones have investigated the potential of integrating higher intensity PA (ie, MVPA) into health promotion efforts. Stair climbing is of higher intensity and can be integrated into daily life [17]. Thus, it could be an efficient way to incorporate MVPA into daily routines, which has shown to result in improved cardiorespiratory fitness and health [18,19]. In view of the potential health benefits and the scarcity of the available evidence, our study investigated the feasibility of using consumer wearables to continuously monitor and promote stair climbing. This study extended the use of static point-of-decision prompts [20] and introduced stair climbing challenges in addition to the more commonly targeted stepping activities.

Despite the convenience of engaging in stepping and stair climbing and the almost effortless and low-cost use of wearables for monitoring such activities, it may not necessarily translate to regular use of these devices anywhere and anytime. Our study revealed that only 60% of participants continued using the trackers, and not all participants contributed valid PA data every day across the 40-day observation period. Although the proportion of valid participants in the control group declined over time, the intervention group demonstrated higher adherence to the use of the trackers, leading to more PA compared with the control group. In terms of stepping, although not significant, the controlled pilot trial suggested greater activity levels in the intervention group. These differences were mainly explained by a decline in stepping among the control group participants, whereas a small proportion of the intervention participants kept or slightly increased their average stepping level over time. After accounting for the correlation between repeated measurements, no significant increase in stepping with respect to weekly challenges was observed. The findings possibly reflect that the provision of self-monitoring alone through the use of wearables may not be sufficient to sustain participants’ interest in PA. A systematic review has suggested that an increase in step count was predicted by behavioral change strategy, such as setting a step goal of 10,000 steps per day beyond just using the wearables [15]. A large-scale factorial randomized controlled trial conducted in Singapore further demonstrated that the use of wearables declined over time and that long-term financial incentives may be needed [49]. Our study provides some initial evidence that a simple, low-cost, and easy to implement team challenge intervention may hold some merits in promoting adherence to the use of wearables. In terms of stair climbing, we observed statistically significant increases in the use of stairs in the intervention group, especially during the second stair climbing challenge. This phenomenon could be partly explained by the fact that climbing stairs may have been seen as a less familiar health promotion target at the time of our study since strategies to promote PA via wearables and smartphones have traditionally focused on stepping goals [15,16]. When the intervention participants were exposed to the stair climbing challenges, they may have gradually become more receptive to the idea, considering the availability of many high-rise buildings and staircases in Singapore. Although we found some positive intervention effects through our use of nonregistered teams, we also recognize that the effects of the team-based intervention might have been different if the team members were friends or family members. Cohesion within each team will likely affect adherence to the intervention and shift the PA profile over time. Moreover, differences across the competing teams will likely be attributed to a complex interplay of social and environmental factors in real-life settings that need to be measured and accounted for in future evaluations. Furthermore, the dosage and timing of interventions delivered in real time require careful consideration. One study suggested that optimizing the number of challenges and feedback messages would potentially improve the engagement and adherence of participants [50]. It is important to ensure that concise messages are presented to targeted users at the right time to minimize cognitive overload [51]. However, in our study, the effects of the number and timing of messages on PA maintenance were not assessed. Future studies are warranted to investigate this question.

In order to deliver effective promotion and monitoring of PA in free-living conditions, delivering meaningful BCTs becomes important. Systematic reviews have identified effective BCTs for health promotion. For instance, self-monitoring, feedback, and goal setting are among the most frequently used BCTs along with the use of commercial wearables in various mHealth strategies [25,50]. Recent studies suggested that strategies based on these BCTs reduced time spent in sedentary behavior [25] and were associated with greater intervention effectiveness for modifying PA and diet behaviors [52]. In addition, increases in intrinsic and extrinsic motivation through the use of reward systems, prompts, and cues have been reported to lead to a more regular use of wearable devices, as well as better adherence and self-management of chronic conditions [50]. Recognizing the growing capabilities of mobile technologies, there are opportunities to perform real-time behavioral assessments and facilitate timely support adapting to individuals’ dynamic behavioral states. A recent systematic review suggested that just-in-time feedback that was goal oriented, actionable, and continuously available significantly improved health behavior [53]. Tailored feedback on users’ current performance [54,55], gamified feedback, such as a leaderboard [31], and user engagement strategies, such as virtual rewards, social connectivity, and adaptive goal setting, have also been found promising in the promotion of PA [56,57]. In addition, studies have also demonstrated an increase in PA levels among the participants who received social incentives compared with participants who exercised alone and performed PA in social settings, such as teamwork and cooperation, compared with competition [58]. In spite of these various promising applications of BCTs in the context of promoting PA in free-living conditions, the potential of technology-enabled, real-time strategies has not been extensively explored [59]. Leveraging selected important BCTs, our study attempted to promote PA in real time through establishing a supportive team-based environment and incorporating feedback, social comparison, reward systems, and cues in addition to self-monitoring. Consistent with the current literature, BCTs may have led to positive stair climbing outcomes favoring the intervention group in our study. Although various BCTs hold potential for improving PA, individual BCTs remain largely underexplored, and it is unclear what combination of BCTs in mHealth interventions may lead to meaningful outcomes [25,60].

This study comprehensively describes the development and initial evaluation of a real-time mHealth team challenge intervention. Our findings suggest that the implementation of the team challenges using an mHealth suite is feasible. First, consumer wearables are readily available to provide a valid estimation of daily stepping and stair climbing activities. Second, mobile internet network connectivity in Singapore enables continuous and real-time monitoring of PA behaviors and allows delivery of in-time feedback about individual and team PA performance. Third, the nonregistering team–based approach adopted in the study helped to eliminate biases related to self-selection into competing teams and demonstrated some improvements in adherence to wearable use and activity levels over time. Fourth, the existing built environment in Singapore and many other metropolitan areas in Asia present opportunities for promoting stair climbing as an additional intervention target beyond traditional stepping-based targets. As such, stair climbing could be readily incorporated into health promotion programs as an effort to promote PA of higher intensity targeting the general adult population. However, we noted that the implementation of such mHealth strategies requires careful considerations. For instance, technical challenges, limited resources for the development or updating of study apps, and variability in smartphone settings prevented installation of the apps’ final version on a larger proportion of participants' phones in our study.

### Strengths and Limitations

This study demonstrated several strengths. First, we validated the wearables for stepping in free-living conditions and also investigated their validity in measuring stair climbing, which has not been studied based on our knowledge. Second, the inference about the effects of the intervention was established from a controlled study design. Third, the wearables were feasible for continuously collecting data on daily stepping and stair climbing activities. However, we also acknowledge several limitations. First, this is an exploratory feasibility study conducted in a single public university, mainly enrolling young to middle-aged educated adults. Thus, the findings have limited generalizability. Second, the intervention duration was short, and future studies will need to investigate long-term outcomes. Third, because of technical challenges in delivering the intervention via participants’ iPhones and a small number of Android phones, we were not able to implement a randomized controlled trial but instead decided to allocate all iPhone users and the affected Android users to the control group. Although this decision may have led to differences between the intervention and control groups, the characteristics of the 2 groups were mostly comparable, and the differences at baseline were adjusted for during the analyses. This circumstance did not reflect the technology affinity of study participants in each group because our study team supported all participants in the installation process. Fourth, we did not collect information on participants’ motivation and users’ experience in relation to mHealth technology, but considering participants’ interest to voluntarily enroll in this study, we assume that the groups’ motivation was comparable. Finally, 40% of recruited participants did not provide valid data for the feasibility study in phase 2. The relatively large dropout rate of participants due to insufficient data hampered the assessment of the intervention’s effectiveness. In this study, we deliberately chose to integrate consumer wearables to monitor study outcomes instead of the more common use of questionnaires or research-grade accelerometers for a few main reasons. For example, validated consumer wearables would likely provide more objective and accurate estimates of stepping and stair climbing than the typical PA questionnaires. These devices also enabled us to monitor outcomes over the entire intervention phase instead of the usual 4 to 7 days of monitoring that is typically the case when using accelerometers. Moreover, using these mobile technologies allowed us to test a less resource-intensive approach to data collection that could be applied in large-scale population health initiatives.

### Conclusions

The consumer wearables integrated into our mHealth suite provided acceptable validity in estimating stepping and stair climbing activities. The mHealth suite was feasible for implementing real-time team challenge interventions. Compared with the controls, the intervention participants performed more stair climbing, which could be introduced as an additional PA promotion target in the context of future mHealth strategies. Methodologically rigorous studies with larger sample size and long-term follow-up are warranted to strengthen this study's findings.

## Appendix

Multimedia Appendix 1 Example of gamification features received by the intervention group.

Multimedia Appendix 2 Comparison of baseline characteristics between non-completers (n=16) and completers (n=24).

## Abbreviations

API: application programming interface
BCT: behavior change technique
GEE: generalized estimating equation
ICC: intraclass correlation coefficient
MdAPE: median of absolute percentage error
mHealth: mobile health
MVPA: moderate-to-vigorous physical activity
PA: physical activity
RR: rate ratio

## References

[1] GBD 2015 Risk Factors Collaborators (2016). Global, regional, and national comparative risk assessment of 79 behavioural, environmental and occupational, and metabolic risks or clusters of risks, 1990-2015: a systematic analysis for the Global Burden of Disease Study 2015. Lancet.

[2] Knuth AG, Hallal PC (2009). Temporal trends in physical activity: a systematic review. J Phys Act Health.

[3] Lee I, Shiroma EJ, Lobelo F, Puska P, Blair SN, Katzmarzyk PT, Lancet Physical Activity Series Working Group (2012). Effect of physical inactivity on major non-communicable diseases worldwide: an analysis of burden of disease and life expectancy. Lancet.

[4] Booth FW, Laye MJ, Lees SJ, Rector RS, Thyfault JP (2008). Reduced physical activity and risk of chronic disease: the biology behind the consequences. Eur J Appl Physiol.

[5] Löllgen H, Böckenhoff A, Knapp G (2009). Physical activity and all-cause mortality: an updated meta-analysis with different intensity categories. Int J Sports Med.

[6] Wen CP, Wai JP, Tsai MK, Yang YC, Cheng TY, Lee M, Chan HT, Tsao CK, Tsai SP, Wu X (2011). Minimum amount of physical activity for reduced mortality and extended life expectancy: a prospective cohort study. Lancet.

[7] Moore SC, Patel AV, Matthews CE, de Gonzalez AB, Park Y, Katki HA, Linet MS, Weiderpass E, Visvanathan K, Helzlsouer KJ, Thun M, Gapstur SM, Hartge P, Lee I (2012). Leisure time physical activity of moderate to vigorous intensity and mortality: a large pooled cohort analysis. PLoS Med.

[8] Sattelmair J, Pertman J, Ding EL, Kohl HW, Haskell W, Lee I (2011). Dose response between physical activity and risk of coronary heart disease: a meta-analysis. Circulation.

[9] Foulds HJ, Bredin SS, Charlesworth SA, Ivey AC, Warburton DE (2014). Exercise volume and intensity: a dose-response relationship with health benefits. Eur J Appl Physiol.

[10] Lee D, Pate RR, Lavie CJ, Sui X, Church TS, Blair SN (2014). Leisure-time running reduces all-cause and cardiovascular mortality risk. J Am Coll Cardiol.

[11] Ekelund U, Steene-Johannessen J, Brown WJ, Fagerland MW, Owen N, Powell KE, Bauman A, Lee I, Lancet Physical Activity Series 2 Executive Committe, Lancet Sedentary Behaviour Working Group (2016). Does physical activity attenuate, or even eliminate, the detrimental association of sitting time with mortality? A harmonised meta-analysis of data from more than 1 million men and women. Lancet.

[12] Donath L, Faude O, Roth R, Zahner L (2014). Effects of stair-climbing on balance, gait, strength, resting heart rate, and submaximal endurance in healthy seniors. Scand J Med Sci Sports.

[13] Meyer P, Kayser B, Kossovsky MP, Sigaud P, Carballo D, Keller P, Martin XE, Farpour-Lambert N, Pichard C, Mach F (2010). Stairs instead of elevators at workplace: cardioprotective effects of a pragmatic intervention. Eur J Cardiovasc Prev Rehabil.

[14] Bellicha A, Kieusseian A, Fontvieille A, Tataranni A, Charreire H, Oppert J (2015). Stair-use interventions in worksites and public settings - a systematic review of effectiveness and external validity. Prev Med.

[15] Bravata DM, Smith-Spangler C, Sundaram V, Gienger AL, Lin N, Lewis R, Stave CD, Olkin I, Sirard JR (2007). Using pedometers to increase physical activity and improve health: a systematic review. J Am Med Assoc.

[16] de Vries HJ, Kooiman TJ, van Ittersum MW, van Brussel M, de Groot M (2016). Do activity monitors increase physical activity in adults with overweight or obesity? A systematic review and meta-analysis. Obesity (Silver Spring).

[17] Teh KC, Aziz AR (2002). Heart rate, oxygen uptake, and energy cost of ascending and descending the stairs. Med Sci Sports Exerc.

[18] Allison MK, Baglole JH, Martin BJ, Macinnis MJ, Gurd BJ, Gibala MJ (2017). Brief intense stair climbing improves cardiorespiratory fitness. Med Sci Sports Exerc.

[19] Gillen JB, Martin BJ, MacInnis MJ, Skelly LE, Tarnopolsky MA, Gibala MJ (2016). Twelve weeks of sprint interval training improves indices of cardiometabolic health similar to traditional endurance training despite a five-fold lower exercise volume and time commitment. PLoS One.

[20] Sloan RA, Haaland BA, Leung C, Müller-Riemenschneider F (2013). The use of point-of-decision prompts to increase stair climbing in Singapore. Int J Environ Res Public Health.

[21] Allais O, Bazoche P, Teyssier S (2017). Getting more people on the stairs: the impact of point-of-decision prompts. Soc Sci Med.

[22] Jacobs RJ, Lou JQ, Ownby RL, Caballero J (2016). A systematic review of eHealth interventions to improve health literacy. Health Informatics J.

[23] Harrington RA, Arena R, Després JP, Ciarochi A, Croll E, Bloch KD, Committee for Scientific Sessions Programmingthe Global Congress on Physical Activity‚ American Heart Association Scientific Sessions 2013 (2015). More than 10 million steps in the right direction: results from the first American Heart Association scientific sessions walking challenge. Prog Cardiovasc Dis.

[24] Schoeppe S, Alley S, van Lippevelde W, Bray NA, Williams SL, Duncan MJ, Vandelanotte C (2016). Efficacy of interventions that use apps to improve diet, physical activity and sedentary behaviour: a systematic review. Int J Behav Nutr Phys Act.

[25] Direito A, Carraça E, Rawstorn J, Whittaker R, Maddison R (2017). mHealth technologies to influence physical activity and sedentary behaviors: behavior change techniques, systematic review and meta-analysis of randomized controlled trials. Ann Behav Med.

[26] Morrison LG, Yardley L, Powell J, Michie S (2012). What design features are used in effective e-health interventions? A review using techniques from Critical Interpretive Synthesis. Telemed J E Health.

[27] Samdal GB, Eide GE, Barth T, Williams G, Meland E (2017). Effective behaviour change techniques for physical activity and healthy eating in overweight and obese adults; systematic review and meta-regression analyses. Int J Behav Nutr Phys Act.

[28] Middleton KR, Anton SD, Perri MG (2013). Long-term adherence to health behavior change. Am J Lifestyle Med.

[29] Duff OM, Walsh DM, Furlong BA, O'Connor NE, Moran KA, Woods CB (2017). Behavior change techniques in physical activity eHealth interventions for people with cardiovascular disease: systematic review. J Med Internet Res.

[30] Looyestyn J, Kernot J, Boshoff K, Ryan J, Edney S, Maher C (2017). Does gamification increase engagement with online programs? A systematic review. PLoS One.

[31] Johnson D, Deterding S, Kuhn K, Staneva A, Stoyanov S, Hides L (2016). Gamification for health and wellbeing: a systematic review of the literature. Internet Interv.

[32] Patel MS, Volpp KG, Rosin R, Bellamy SL, Small DS, Fletcher MA, Osman-Koss R, Brady JL, Haff N, Lee SM, Wesby L, Hoffer K, Shuttleworth D, Taylor DH, Hilbert V, Zhu J, Yang L, Wang X, Asch DA (2016). A randomized trial of social comparison feedback and financial incentives to increase physical activity. Am J Health Promot.

[33] Patel MS, Asch DA, Rosin R, Small DS, Bellamy SL, Eberbach K, Walters KJ, Haff N, Lee SM, Wesby L, Hoffer K, Shuttleworth D, Taylor DH, Hilbert V, Zhu J, Yang L, Wang X, Volpp KG (2016). Individual versus team-based financial incentives to increase physical activity: a randomized, controlled trial. J Gen Intern Med.

[34] Kullgren JT, Troxel AB, Loewenstein G, Asch DA, Norton LA, Wesby L, Tao Y, Zhu J, Volpp KG (2013). Individual- versus group-based financial incentives for weight loss: a randomized, controlled trial. Ann Intern Med.

[35] Gollwitzer PM (1999). Implementation intentions: strong effects of simple plans. Am Psychol.

[36] Michie S, Ashford S, Sniehotta FF, Dombrowski SU, Bishop A, French DP (2011). A refined taxonomy of behaviour change techniques to help people change their physical activity and healthy eating behaviours: the CALO-RE taxonomy. Psychol Health.

[37] Coughlin SS, Stewart J (2016). Use of consumer wearable devices to promote physical activity: a review of health intervention studies. J Environ Health Sci.

[38] Feehan LM, Geldman J, Sayre EC, Park C, Ezzat AM, Yoo JY, Hamilton CB, Li LC (2018). Accuracy of Fitbit devices: systematic review and narrative syntheses of quantitative data. JMIR Mhealth Uhealth.

[39] Gorny AW, Liew SJ, Tan CS, Müller-Riemenschneider F (2017). Fitbit charge HR wireless heart rate monitor: validation study conducted under free-living conditions. JMIR Mhealth Uhealth.

[40] Straiton N, Alharbi M, Bauman A, Neubeck L, Gullick J, Bhindi R, Gallagher R (2018). The validity and reliability of consumer-grade activity trackers in older, community-dwelling adults: a systematic review. Maturitas.

[41] Degroote L, de Bourdeaudhuij I, Verloigne M, Poppe L, Crombez G (2018). The accuracy of smart devices for measuring physical activity in daily life: validation study. JMIR Mhealth Uhealth.

[42] Farina N, Lowry RG (2018). The validity of consumer-level activity monitors in healthy older adults in free-living conditions. J Aging Phys Act.

[43] Chen R, Gong J (2018). Can self selection create high-performing teams?. J Econ Behav Organ.

[44] Edwards EA, Lumsden J, Rivas C, Steed L, Edwards LA, Thiyagarajan A, Sohanpal R, Caton H, Griffiths CJ, Munafò MR, Taylor S, Walton RT (2016). Gamification for health promotion: systematic review of behaviour change techniques in smartphone apps. BMJ Open.

[45] Tudor-Locke C, Barreira TV, Schuna JM (2015). Comparison of step outputs for waist and wrist accelerometer attachment sites. Med Sci Sports Exerc.

[46] Trost SG, McIver KL, Pate RR (2005). Conducting accelerometer-based activity assessments in field-based research. Med Sci Sports Exerc.

[47] HealthHub.

[48] Lee JA, Williams SM, Brown DD, Laurson KR (2015). Concurrent validation of the Actigraph gt3x+, Polar Active accelerometer, Omron HJ-720 and Yamax Digiwalker SW-701 pedometer step counts in lab-based and free-living settings. J Sports Sci.

[49] Finkelstein EA, Haaland BA, Bilger M, Sahasranaman A, Sloan RA, Nang EEK, Evenson KR (2016). Effectiveness of activity trackers with and without incentives to increase physical activity (TRIPPA): a randomised controlled trial. Lancet Diabetes Endocrinol.

[50] Sardi L, Idri A, Fernández-Alemán JL (2017). A systematic review of gamification in e-Health. J Biomed Inform.

[51] Nahum-Shani I, Smith SN, Spring BJ, Collins LM, Witkiewitz K, Tewari A, Murphy SA (2018). Just-in-Time Adaptive Interventions (JITAIs) in mobile health: key components and design principles for ongoing health behavior support. Ann Behav Med.

[52] Michie S, Abraham C, Whittington C, McAteer J, Gupta S (2009). Effective techniques in healthy eating and physical activity interventions: a meta-regression. Health Psychol.

[53] Schembre SM, Liao Y, Robertson MC, Dunton GF, Kerr J, Haffey ME, Burnett T, Basen-Engquist K, Hicklen RS (2018). Just-in-time feedback in diet and physical activity interventions: systematic review and practical design framework. J Med Internet Res.

[54] Martin SS, Feldman DI, Blumenthal RS, Jones SR, Post WS, McKibben RA, Michos ED, Ndumele CE, Ratchford EV, Coresh J, Blaha MJ (2015). mActive: a randomized clinical trial of an automated mHealth intervention for physical activity promotion. J Am Heart Assoc.

[55] Walsh JC, Corbett T, Hogan M, Duggan J, McNamara A (2016). An mHealth intervention using a smartphone app to increase walking behavior in young adults: a pilot study. JMIR Mhealth Uhealth.

[56] Poirier J, Bennett WL, Jerome GJ, Shah NG, Lazo M, Yeh H, Clark JM, Cobb NK (2016). Effectiveness of an activity tracker- and internet-based adaptive walking program for adults: a randomized controlled trial. J Med Internet Res.

[57] Adams MA, Hurley JC, Todd M, Bhuiyan N, Jarrett CL, Tucker WJ, Hollingshead KE, Angadi SS (2017). Adaptive goal setting and financial incentives: a 2 × 2 factorial randomized controlled trial to increase adults' physical activity. BMC Public Health.

[58] Chen Y, Pu P (2014). HealthyTogether: Exploring Social Incentives for Mobile Fitness Applications. Proceedings of the Second International Symposium of Chinese CHI.

[59] O'Reilly GA, Spruijt-Metz D (2013). Current mHealth technologies for physical activity assessment and promotion. Am J Prev Med.

[60] Eckerstorfer LV, Tanzer NK, Vogrincic-Haselbacher C, Kedia G, Brohmer H, Dinslaken I, Corcoran K (2018). Key elements of mHealth interventions to successfully increase physical activity: meta-regression. JMIR Mhealth Uhealth.

