# Supplementary material for: A Mobile Health Team Challenge to Promote Stepping and Stair Climbing Activities: Exploratory Feasibility Study
Source: JMIR Mhealth Uhealth. 2020 Feb 4;8(2):e12665. doi: 10.2196/12665 (PMC7055777; doi:10.2196/12665)
Supplement: Multimedia Appendix 1 [file mhealth_v8i2e12665_app1.docx]

**Appendix-1**

**Announcement of weekly team-challenge (for Monday – Friday)**


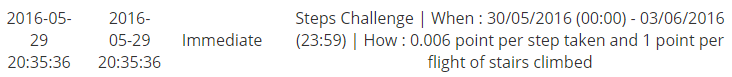


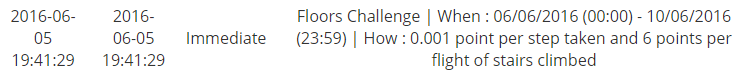


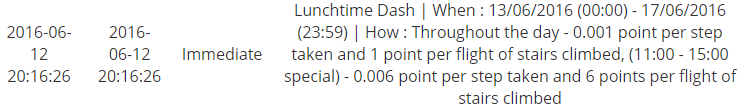


**Announcement of the leading team during mid-week (Wednesday)**


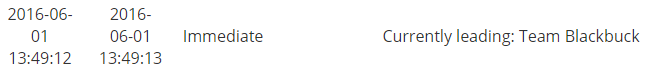


**Announcement of the winning team at the end of each challenge week**


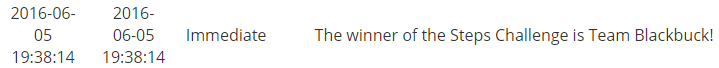


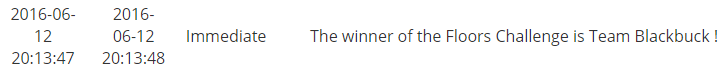

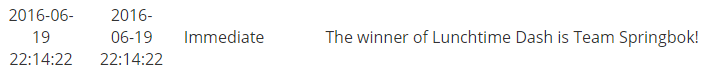


Figure A-1: Example of push notifications, motivational messages delivered via study app

**Leaderboard**


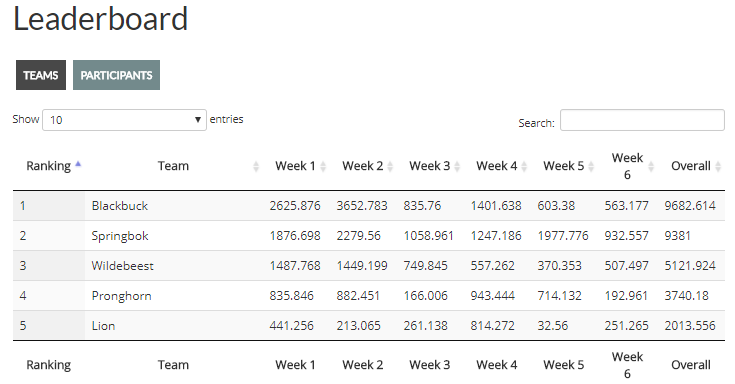


Figure A-2: Example of leaderboard keeping track of weekly scores and ranking of team
