# Supplementary material for: A Mobile Health Team Challenge to Promote Stepping and Stair Climbing Activities: Exploratory Feasibility Study
Source: JMIR Mhealth Uhealth. 2020 Feb 4;8(2):e12665. doi: 10.2196/12665 (PMC7055777; doi:10.2196/12665)
Supplement: Multimedia Appendix 2 [file mhealth_v8i2e12665_app2.docx]

**Appendix-2**

Table A- 1: Comparison of baseline characteristics between non-completers (n=16) and completers (n=24)

| **Baseline characteristics** | | | **Full sample (N=40)** | | | | | |
| --- | --- | --- | --- | --- | --- | --- | --- | --- |
|  |  |  | **Non-completer**  **(n=16)** | | **Completer^a^**  **(n=24)** | | **Prevalence ratio**  **(95%CI) *P* value^b^** | |
| **Age, median (25th – 75th percentile)** | | | 24.5 (23-27) | | 28 (23-32.5) | | 0.272^c^ | |
| **Age, n(%)** | ≤25 years | 9 (56.3) | | 10 (41.7) | | 1.00 [Ref.] | |  |
|  | >25 years | 7 (43.7) | | 14 58.3) | | 1.27 (0.75, 2.14) *P*=.52 | |  |
| **Gender, n (%)** | Female | 5 (31.3) | | 15 (62.5) | | 1.00 [Ref.] | |  |
|  | Male | 11 (68.7) | | 9 (37.5) | | 0.60 (0.35, 1.03) *P* =.11 | |  |
| **Ethnicity, n (%)** | Chinese | 11 (68.7) | | 20 (83.3) | | 1.00 [Ref.] | |  |
|  | Non-Chinese | 5 (31.3) | | 4 (16.7) | | 0.69 (0.32, 1.50) *P* =.44 | |  |
| **Education, n (%)** | Secondary^d^ | 4 (25.0) | | 7 (29.2) | | 1.00 [Ref.] | |  |
|  | Tertiary or above | 12 (75.0) | | 17 (70.8) | | 0.92 (0.54, 1.58) *P* =1.0 | |  |
| **Work, n (%)** | Studying | 12 (75.0) | | 13 (54.2) | | 1.00 [Ref.] | |  |
|  | Working | 4 (25.0) | | 11 (45.8) | | 1.41 (0.87, 2.29) *P* =.32 | |  |
| **Marital status, n (%)** | Not married | 14 (87.5) | | 19 (79.2) | | 1.00 [Ref.] | |  |
|  | Married | 2 (12.5) | | 5 (20.8) | | 1.24 (0.71, 2.16) *P* =.68 | |  |
| ^a^Completer is defined as participants who were valid at both baseline and ending phase and contributed complete exposure data.  ^b^Prevalence ratio, 2-sided Fisher’s exact.  ^c^Wilcoxon rank sum tests (medians).  ^d^Secondary educational level included participants who completed A-level or attended polytechnic school | | | | | | | | |
